# Tooth Cementum Thickness as a Method of Age Estimation in the Forensic Context

**DOI:** 10.3390/biology11050784

**Published:** 2022-05-21

**Authors:** Emanuela Gualdi-Russo, Ilaria Saguto, Paolo Frisoni, Margherita Neri, Natascia Rinaldo

**Affiliations:** 1 Department of Neuroscience and Rehabilitation, University of Ferrara, 44121 Ferrara, Italy; ilaria.saguto@unife.it; 2 Department of Medical Sciences, University of Ferrara, 44121 Ferrara, Italy; paolo.frisoni@unife.it

**Keywords:** age estimation, tooth root, cementum thickness, forensic anthropology

## Abstract

**Simple Summary:**

In a forensic context, it may be necessary to estimate the age at death of an individual from his or her skeletal remains. The age at death constitutes an essential identification parameter, along with sex, ancestry, stature, and a few other biological characteristics that can help to trace the biological profile of the deceased. Although there is an extensive number of works in the literature on this topic, traditional anthropological methods encounter considerable difficulty in estimating the age of an adult based on skeletal remains. This difficulty increases with aging mainly due to the overlapping of pathological processes. In this study, we tested the reliability of a relatively simple histological methodology to estimate an individual′s age based on tooth roots cementum and developed new equations from individuals with known age and sex. In conclusion, our results confirm the importance of teeth in the estimation of age, suggesting that the proposed method may be effective, especially for younger individuals.

**Abstract:**

Estimating age at death is a key element in the process of human identification of skeletal remains. The interest in dental cementum stems from its increase in thickness throughout life and, at the same time, from the fact it should not be affected by remodeling processes. Since the age assessment is particularly difficult in adults when using traditional anthropological methods on the skeleton, we tested a dental method based on maximum cementum thickness and developed new regression equations. We microscopically analyzed the histological sections of dental roots from a sample of 108 permanent teeth with known age and sex. Age at the time of dental extraction was in the range of 18–84 years. Our findings show that there were no differences in thickness between sexes, dental arch, and mono- and pluriradicular teeth. Separate regression equations were developed for individuals in the whole age range and individuals under 45 years. The equations were then tested on a hold-out sample from the same Mediterranean population demonstrating higher reliability for the equation developed for those under 45. Conversely, due to the increased error in age estimation in individuals over 45, this method should be used with caution in the forensic context when skeletal remains presumably belong to elderly individuals.

## 1. Introduction

Estimation of age at death is a key part of the biological profile in the case of unknown human skeletal remains. However, the choice of the most appropriate anthropological method and the uncertainty of age-at-death estimation are still highly debated issues [1]. Both skeletal and dental methods may be applied. In adults, skeletal age estimation is generally based on indicators involved in the process of bone resorption, deposition, and remodeling. In particular, cranial sutures obliteration, pubic symphysis, auricular surface, and sternal rib end are the most commonly employed methods (among others, see References [2,3,4,5,6,7]). More recently, other methods based on a Bayesian approach have been developed, thus minimizing the bias caused by inter-population variation [8,9]. The range of estimated age is narrower in the young and wider in the elderly [10].

Teeth are more resistant than other tissues to decay and degradation and, in some cases, represent the only useful elements of identification. Therefore, methods for age estimation based on teeth have also been developed and applied. Dental age-estimation methods include, among others, tooth cementum annulation (TCA), the Lamendin method, and aspartic acid racemization [11,12,13]. Even in the case of dental methods, there is much debate as to which method allows for the most accurate estimation of the chronological age of the individual [14,15].

Cunha et al. [1] also emphasized that the best methods are those that are appropriate for a particular forensic context; are practical, easy to use, relatively fast, and inexpensive; and have been successfully proven in several populations. Consistently with this assumption, we deemed it important to test a dental method that was simpler and faster than TCA counting or aspartic acid racemization and that seemed to have good potential: the dental cementum thickness.

Although the composition of cementum is chemically similar to bone, it has two more favorable characteristics for the age-of-death assessment: (1) it is not affected by the remodeling bony processes, and (2) its apposition is continuous throughout an individual’s life [16]. These same arguments would seem to support, in general, the use of cementochronology, which is based on the counting of incremental lines in tooth root cementum to assess the age at death of an individual. However, several factors, such as technical difficulties and the variability in the apposition of incremental lines, have restricted the utilization of this method [17,18]. An alternative approach appears to be the direct measurement of cementum thickness, as it is found to increase with age [19], with the strongest correlations between age and dental cementum at one-third of the tooth root [20].

Hence, the present study was performed to evaluate the association between cementum thickness and age and to provide new regression equations for age prediction from cementum thickness measured in a sample of the Italian population with known age and sex.

## 2. Materials and Methods

Our sample consists of 108 permanent teeth extracted from 108 individuals due to periodontal reasons. Tooth extractions were carried out in the period 2015–2019 on Italian adults of both sexes in some clinics located in the Emilia-Romagna region (Italy). Teeth broken during extraction or with tooth root disease (dental root caries) were excluded from the study.

At the time of extraction, the individual′s age and sex were recorded, but the person’s anonymity was maintained. Sample ages were within the range of 18–84 years.

No preference was given either to tooth type or the dental arch. Overall, the composition of the sample by tooth type was as follows: 9.3% incisors, 4.6% canines, 11.1% premolars, and 75.0% molars. Before sectioning, a code number was given to each tooth that was then photographed. Using a tooth as a unit, only one tooth for a single individual was considered in this study.

Although cementum apposition is continuous throughout life, its rate varies (slowing with aging), thus inducing a more difficult age estimate. It is known that there is a 1/3 decrease in cementum apposition with advancing age after the age of 60 [21]. The threshold beyond which such apposition begins to slow down considerably, showing irregularities, is likely to be between 40 and 50/55 years of age [22,23]. In a previous study [18], we observed errors greater than 10 years in age estimation by TCA counting in subjects older than 43.4 years. In consideration of the previous literature, in this study, we used a conventional cutoff of 45 years to separate Early Adulthood and Early Middle Age from Late Middle Age and Late Adulthood [24]. Other anthropological classifications to estimate age from the skeleton also resort to these same age classes [2].

The procedure followed for histological preparation has been described in detail previously [18]. After decalcification, roots were trimmed on mesial and distal aspects parallel to the midsagittal line of the root, giving a landmark site for parallel longitudinal sectioning in the buccolingual direction, as suggested by the literature [19,20]. After paraffin-embedding, the teeth were cut into 10 µm sections at one-third of the root length from the apex [20]. Then we identified the site that showed the maximum acellular cementum thickness all along the perimeter of the tooth slice (excluding the area of cellular cementum where the roots joint in the pluriradicular teeth). We collected this measurement for each tooth by using Optika Microscope B-500Ti at 10× and 40× magnification and real-time image Moticon Plus 3.0™ software for digital image enhancement.

Descriptive statistics were used to present and interpret the data. Given the reduced speed of cementum apposition with aging, we performed comparisons between the group of subjects aged <45 years versus the group ≥ 45 years. The Kolmogorov–Smirnov test (K–S) was applied to assess the normality of variable distribution. Cementum thickness showed a non-normal distribution (K–S d = 0.1477, p < 0.05) in the total sample. However, a more in-depth analysis for age groups (< and ≥ 45 years) showed that the distribution of this variable was normal up to an age < 45 years (K–S d = 0.09528, *p* > 0.20). When the distribution of data was normal, we performed the statistical analysis, using parametric statistics (linear regression analysis, Pearson’s r, and R^2^). Otherwise, we used non-parametric statistics (Mann–Whitney U test, Wilcoxon Matched-Pairs Test, non-linear regression analysis, and Spearman’s ρ).

Before further processing the data, however, we randomly selected a subset of 18 individuals (9 < 45 years; 9 ≥ 45 years) that were used for cross-validation (hold-out sample). Their cementum-thickness data were not included in the database that we used to generate the new regression equations.

The thickness–age (tooth-age according to Reference [20]) was computed for each tooth by using the individual known age minus the mean age of tooth eruption according to Reference [25]. To develop the prediction model of a variable with normal distribution, we computed the linear regression for the variable relative to the age, coefficients of correlation (r), and determination (R^2^). In the case of non-normal distribution, we identified the regression model (logarithmic) that was found to give plausible data to describe the mathematical association between the variable and the age, and Spearman’s correlation coefficient (ρ). Standard errors of the estimate (SEE) were also computed. Given the significant differences between groups of different ages in cementum thickness, we selected 74 individuals as “study sample” from the 90 of the total sample (overall sample minus hold-out sample), balancing the number of individuals from the <45 years old group and the ≥45 years old group. In particular, regression equations were developed for both the under-45 group and the study sample (combined age sample) comprising the same number of those under 45 (*n* = 37) and those over 45 (*n* = 37). The latter were randomly selected from the 53 subjects in the older group.

The accuracy of the proposed new regression equations was assessed by applying the formulas derived from the study group to the hold-out sample, and the estimated age was compared with the known age of these individuals. Bias (Δ) of estimated age, as the difference between the estimated age and chronological age (Δ = Ageest – Agechronol), was computed in the hold-out sample. For comparison purposes, we calculated on the same hold-out sample the percent bias of prediction (%Δ), as follows: (|Ageest – Agechronol|/Agechronol) × 100. 

Data analysis was performed by using STATISTICA for Windows (version 11.0, StatSoft, Tulsa, OK, USA).

The level of statistical significance was set at *p* ≤ 0.05.

## 3. Results

Table 1 shows the main features of the whole sample examined. We had a fairly wide range of ages in the current sample (18–84 years; mean age = 48.2 ± 19.7 years), including both males and females, single and multiple tooth roots, and upper and lower dental arch. The maximum cementum thickness ranged from 54.9 to 341.2 µm (mean 164.1 ± 67.3 µm), with an average thickness age of 35.4 years (SD = 21.5). Teeth with multiple roots and those extracted from the maxilla were found to be prevalent in the sample.

Table 2 shows descriptive statistics of the sample separately by sex, age group, dental arch, and the number of tooth roots. The statistical comparison by sex, dental arch, and tooth root revealed no significant difference in cementum thickness, in contrast to the comparison by age group: individuals younger than 45 years had, on average, significantly less cementum thickness than older individuals.

**Figure 1:**
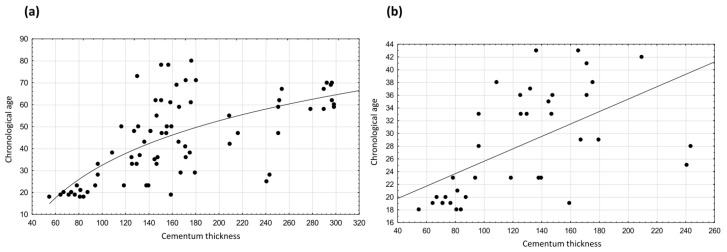
Scatterplots showing the relationship between dental cementum thickness and chronological age: (**a**) in the study sample (*n* = 74) and (**b**) in the <45 years age group (*n* = 37).

After removing the 18 cases selected for the hold-out sample and after balancing the number of individuals under 45 and over 45 in the study sample, we drew the graphs shown in Figure 1. In particular, Figure 1 presents the graph of dispersion of chronological age on cementum thickness with the regression curve or line drawn in the study sample (a) and in the under-45 age group (b), respectively.

Table 3 shows the regression equations developed separately for the study sample and the under-45 age group. Relationships between chronological age and cementum thickness were statistically significant in both evaluations. In contrast to the study sample and the under-45 age group, no significant association was found between cementum thickness and age in subjects over 45 years of age (Spearman rho = 0.269, *p* > 0.05). Therefore, no predictive equation was calculated for the ≥45 age group.

The values of the standard error of estimate (SEE), which indicates the error that may occur in age estimation, are much lower (less than half) in the <45 age group than in the study sample, as expected, given the wide age range of the latter. Based on the coefficient of determination R^2^ calculated in the under-45 age group, dental cementum thickness accounts for 32% of the variation in age of the younger group.

The results reported in Table 4 show that the comparison between the chronological ages and the estimated ages that were obtained by applying both regression equations did not display any significant differences in the hold-out sample. Although age estimation with the linear regression equation applied to younger individuals resulted, as expected, in mean values characterized by less variability (SD) and inaccuracy (Δ, %Δ) compared to the hold-out sample with individuals of all chronological ages, both equations showed good agreement, on average, between chronological and predicted ages, with an underestimate of one year in the youngest and an overestimate of nearly three years in the total hold-out sample.

As a further check, we applied Equation (1) to the subsample <45 years old and obtained an age estimate that was not statistically significant (*p* >.05) compared to the chronological age (39.6 ± 12.4 years vs. 30.3 ± 8.5 years). However, a bias of 9.3 years (47.6 %) resulted in this estimate. Finally, the last possible application of Equation (1) to the >45 age group showed a significant difference (*p* < 0.05) between estimated age and chronological age (47.5 ± 9.0 years vs. 62.1 ± 12.7 years) with a bias of 14.7 years (21.6%).

## 4. Discussion

This study provides support for the use of dental cementum thickness in the age estimation of young adults. In particular, two new mathematical models have been developed for predicting individual age from maximum cementum thickness, the first (Equation (1)) based on the whole study sample (wide age range) and the second (Equation (2)) on the study subsample under 45 years of age.

Age estimation is of crucial importance in forensics, as it is a fundamental part of the biological profile of the individual required for his/her identification from skeletal remains. Several methods for estimating the age in adults from the teeth have been proposed to complete the skeletal analysis. Although measuring the total cementum thickness was one of the first methods used for human age estimation from teeth [26], studies related to tooth cementum annulations have subsequently prevailed. This preference may have arisen from the belief that it is simpler and more precise to directly obtain the age estimation by adding the incremental line count to the age of tooth eruption rather than measuring the cementum thickness and employing appropriate regression equations to obtain the age estimate. However, even TCA counting has some limitations, as the bands are not always easy to identify, especially in the elderly [18,27], and the accuracy of the method depends on several factors, such as histological preparation techniques, age of the individual, and tooth type and preservation [28,29]. This study addressed the problem of age estimation by using the maximum thickness of dental cementum: following Cunha′s recommendation [1], we performed age estimation by a relatively simpler and faster method than TCA. Moreover, this method allows us to estimate the age at death even if the bands are not observable for TCA. 

Due to its continuous apposition, cementum thickness represents, indeed, a potentially age-estimating tissue [30]. The cementum thickness differs concerning the region of the root, with a maximum at the apex and a minimum at the cementum–enamel junction (CEJ) [16,20,31], probably due to the influence of compensatory eruption and attrition as reported by Reference [20]. In this case, we decided to consider the maximum cementum thickness at 1/3 of the root length, because cementum thickness at this site is believed to be a more accurate predictor of age than cementum thickness at the apex [20,32], although not unanimously [33]. On the other hand, a preferred side of the dental arch was not selected, since no difference was found between right and left teeth [20,34]. Univariate analyses conducted in the examined sample showed that there were no significant differences in maximum cementum thickness between sexes, upper and lower dental arches, and between single-rooted and multi-rooted teeth, while thickness was significantly greater in the older subsample than in the younger one, consistently with the known trend that leads to cementum thickening with aging. This generally confirms the results of other studies in the literature apex [32,33], although Solheim [20] reported greater thicknesses in the male sex and young people.

To date, only a few studies have addressed age estimation directly from the measurement of dental cementum thickness [16,19,20,22,30,32,33]. Moreover, only the last three studies cited have developed equations for age estimation by using tooth cementum thickness. Among these, Nitzan et al. [19] calculated the linear regression of thickness at the apex and in central regions of the tooth root with aging. Solheim [20] developed multiple regression equations separately for each type of tooth. Finally, two different nonlinear regression equations were proposed to estimate age from cementum thickness at the apex and one-third of root length from the apex (vestibular and lingual sides), distinguishing according to the tooth type [33]. After analyzing several methods and variables for estimating age from teeth, Soomer et al. [14] emphasized the importance of a simple methodology for forensic use and the greater reliability of the methods for sectioned teeth in comparison with those for intact teeth. According to another study [15], when comparing different methods of age estimation based on teeth, the method of Johanson [35] with six variables for each tooth (attrition, secondary dentin, cementum apposition, periodontal recession, root resorption, and root translucency) was considered to be the best for the dental age estimation of war victims exhumed from the mass graves in Croatia.

In this study, we developed two new equations to estimate the age of unknown individuals based on maximum dental cementum thickness from 18 years of age to over 80 and from 18 to under 45, respectively. From a procedural standpoint, if an initial assessment with multiple anthropological methods, such as the auricular surface of the ilium, pubic symphysis, and the sternal end of the fourth rib [36,37,38], suggested the individual belonged to the young/early middle adult group, we recommend the application of the linear regression equation developed for this age group (Equation (2)). In the case that no age assumption can be made or older age is assumed, the logarithmic regression equation is more suitable (Equation (1)). Both proposed equations seem effective at predicting age, as reflected by the significant association between maximum cementum thickness and age in the sample with known age. Furthermore, the application of the equations on the hold-out sample showed that there were no significant differences between predicted and chronological age when Equation (1) was applied to the entire hold-out sample or when Equation (2) was applied to the under-45 individuals of the hold-out sample. However, we found that Δ and %Δ were greater in the entire hold-out sample, on whom the nonlinear regression equation was applied than in the younger hold-out subsample. For individuals under 45, the results obtained show lower values of inaccuracy with an underestimate of one year in age, on average. The attempted extension of Equation (1) to other age groups of the hold-out sample gave acceptable results only in individuals < 45 years old, but was ineffective in predicting age in individuals ≥ 45 years of age of the hold-out sample: inaccuracy exceeded, on average, 10 years of underestimation in age, regarded as a threshold value in the forensic field [39]. This may be linked to the fact that local resorption at the cementum surface and subsequent apposition could be observed with aging, thus increasing the irregularity of the cemental surface [40]. More broadly, the difficulty in estimating the age of elderly subjects is consistent with the general trend of anthropological methods showing an increase in the error involved in age estimation, especially beyond the fifth decade [41]. In brief, our results suggest that the application of Equation (2) in young people is sufficiently reliable in predicting age from dental cementum thickness, whereas Equation (1) is to be applied with extreme caution and, possibly, as an additional method for estimating the age of unknown individuals. In all cases, as already highlighted in the literature decade [42,43], multiple methods or indicators, rather than single ones, should be applied in the identification process.

Restricting our consideration to the association between chronological age and cementum thickness in the young group, we found that the thickness accounted for only 32% of the variability in chronological age based on the coefficient of determination. In interpreting the variability in individual age by cementum thickness (R^2^), an element often overlooked in the literature must be taken into account: the estimate of the age does not concern the chronological age, but the biological age. While chronological aging concerns only the passage of time, biological aging relates to deteriorative processes affecting different bodily structures and functions [44,45]. In particular, several processes occur with aging in the skeleton, leading to bone resorption, deposition, and remodeling, and in dental histology and morphology features, leading to changes in hard (cementum, enamel, and dentin) and soft (dental pulp) tissues of the tooth with consequent tooth wear, enamel brittleness, increased root caries incidence, and pulp cavity constriction [46,47,48]: these changes may differ in the patterns and timing of occurrence among individuals and populations resulting in a different biological age for individuals of the same chronological age [10,30]. Endogenous and exogenous factors can affect the biological aging process, becoming a potential source of bias in age estimation [49,50,51]. In old age, moreover, pathological conditions can intervene to further complicate the picture [10]. Although there is a significant association between chronological age and biological age, it should therefore be clearly stated that all the analyses finalized to the estimation of age on the skeleton and the teeth always refer to biological age. Most likely, the bias between actual and estimated age depends mainly on this factor. Moreover, in analogy to dental annulations [52,53], we cannot exclude that the thickness of the dental cementum may be affected also by other factors occurring during people′s lives, especially those involving calcium metabolism [54].

The strengths of the study include the relative simplicity of detection of this quantitative indicator of age (maximum cementum thickness) compared with other teeth indicators (for example, TCA and aspartic acid racemization) and that teeth are less affected by environmental factors than degenerative processes of the skeleton (morphological traits evaluation). In addition, an appreciable feature of this indicator is that it seems to be not affected by sex (as reported above), which is an important aspect when dealing with unknown human remains in forensics. Moreover, in contrast to other studies conducted on human remains from past epochs, this study refers to a contemporary Mediterranean population, obtaining results that are applicable in a forensic context. However, the proposed equations should also be tested in other populations. Our study is not without weaknesses. The histological technique used falls under destructive methods. We did not assess inter- and intra-observer variability in the measurement of dental cementum thickness. The teeth analyzed in this study were not balanced in terms of tooth type (molars were prevalent). However, no significant differences were found in the thickness of single- and multiple-rooted teeth. Finally, the teeth examined were all extracted for therapeutic reasons. However, studies performed on TCA show that dental pathologies, with the exclusion of deep pathologies, do not affect the dental cementum metabolism [55,56].

## 5. Conclusions

The continuous apposition of cementum during the aging process makes it a potential predictor of biological age. Therefore, we analyzed a sample of individuals from a wide range of chronological ages and developed regression equations based on maximum cementum thickness. These equations can be applied to estimate biological age in the Mediterranean population on both sexes, using teeth of any type. However, as is true for the generality of methods used to predict age from the skeleton, an increase in variability with aging and a greater error involved in estimating age, especially after age 45, have been found. The bias between actual and estimated age may depend mainly on the difference between chronological and biological age. Therefore, while the linear regression equation developed for younger subjects is applicable with confidence, great caution is suggested in the application of the logarithmic regression equation to the elderly and, in any case, above 45 years of age.

Our research demonstrated that teeth provide useful indications in age estimation also in adults: in the forensic field, the new proposed equations can be applied to unidentified skeletal remains during biological profiling.

## Figures and Tables

**Table 1:** Number of teeth by sex, age, tooth root, and dental arch in the overall sample (*n* = 108).

Variables	*n*	%
Sex		
Males	46	42.6
Females	62	57.4
Age		
<45 years	46	42.6
≥45 years	62	57.4
Tooth root		
Single	27	25.0
Multiple	81	75.0
Dental arch		
Maxilla	81	75.0
Mandibula	27	25.0

**Table 2:** Descriptive statistic data of the overall sample (*n* = 108). The *p*-value shows the difference for cementum thickness between subsamples by the Mann–Whitney U test.

	Cementum Thickness (µm)			
	*n*	Mean	SD	*p*
Sex				0.6591
Males	46	167.0	66.9	
Females	62	161.9	68.1	
Age				**<0.00001**
<45 years	46	128.4	50.3	
≥45 years	62	190.5	66.4	
Dental arch				0.8900
Maxilla	81	164.6	69.0	
Mandibula	27	162.3	63.2	
Tooth root				0.5607
Single	27	176.6	74.6	
Multiple	81	159.9	64.7	

**Table 3:** New equations for the prediction of chronological age (Y) by maximum cementum thickness (X), using the study sample (age range: 18–84) and under-45 age group.

Parameter	Age Group
	**Age 18–84** (*n* = 74)
1-Logarithmic regression equation	Y= −102.07 + 67.24 × LOG (X)
SEE	17.2
Spearman rho	0.670
*p*	<0.05
	**Age < 45** (*n* = 37)
2-Linear regression equation	Y = 15.87 + 0.097 × X
SEE	7.0
Pearson r	0.568
R^2^	0.323
*p*	0.0002

**Table 4:** Comparisons between chronological and estimated age by Wilcoxon Matched-Pairs Test. The predicted age was estimated by logarithmic regression equation (Y) in the hold-out sample and by linear regression equation in the hold-out subsample <45 years.

Hold-Out Sample		Known Age (years)				Predicted Age (years)					Δ		%Δ	
	Equation	Range	Mean	Median	SD	Range	Mean	Median	SD	p	Mean	SD	Mean	SD
Total (*n* = 18)	1	18–84	46.2	44.5	19.4	17–65	43.6	41.8	11.3	0.61	2.7	17.9	34.6	29.4
<45 years (*n* = 9)	2	18–41	30.3	33.0	8.5	21–40	29.3	27.6	5.6	0.95	−1.0	8.7	21.8	17.1

## Data Availability

The databases used and/or analyzed during the current study are available from the corresponding authors upon reasonable request.
